# Soft-Sensor for Class Prediction of the Percentage of Pentanes in Butane at a Debutanizer Column

**DOI:** 10.3390/s21123991

**Published:** 2021-06-09

**Authors:** Iratxe Niño-Adan, Itziar Landa-Torres, Diana Manjarres, Eva Portillo, Lucía Orbe

**Affiliations:** 1 TECNALIA, Basque Research and Technology Alliance (BRTA), 48160 Derio, Spain; diana.manjarres@tecnalia.com; 2 Department of Automatic Control and Systems Engineering, Faculty of Engineering, University of the Basque Country UPV/EHU, 48013 Bilbao, Spain; eva.portillo@ehu.es; 3 Petronor Innovación S.L, 48550 Muskiz, Spain; itziar.landa@repsol.com (I.L.-T.); lucia.orbe@repsol.com (L.O.)

**Keywords:** pentanes, classification, autoML, soft-sensor, normalisation, feature weighting

## Abstract

Refineries are complex industrial systems that transform crude oil into more valuable subproducts. Due to the advances in sensors, easily measurable variables are continuously monitored and several data-driven soft-sensors are proposed to control the distillation process and the quality of the resultant subproducts. However, data preprocessing and soft-sensor modelling are still complex and time-consuming tasks that are expected to be automatised in the context of Industry 4.0. Although recently several automated learning (autoML) approaches have been proposed, these rely on model configuration and hyper-parameters optimisation. This paper advances the state-of-the-art by proposing an autoML approach that selects, among different normalisation and feature weighting preprocessing techniques and various well-known Machine Learning (ML) algorithms, the best configuration to create a reliable soft-sensor for the problem at hand. As proven in this research, each normalisation method transforms a given dataset differently, which ultimately affects the ML algorithm performance. The presented autoML approach considers the features preprocessing importance, including it, and the algorithm selection and configuration, as a fundamental stage of the methodology. The proposed autoML approach is applied to real data from a refinery in the Basque Country to create a soft-sensor in order to complement the operators’ decision-making that, based on the operational variables of a distillation process, detects 400 min in advance with 98.925% precision if the resultant product does not reach the quality standards.

## 1. Introduction

Refineries are complex industrial systems that transform crude oil into more valuable subproducts, i.e., Liquefied Petroleum Gas (LPG), gasoline or petrol, kerosene, jet fuel, diesel oil and fuels oils. One of their primary concerns is to ensure high-quality final subproducts that meet the rigorous government regulations to achieve the maximum profit for commercialising them. In this context, due to the advances in sensing, easy-to-measure variables are continuously monitored, and several data-driven soft-sensors are proposed to control the distillation process and the quality of the resultant subproducts. In this research line, there are several works for monitoring and controlling different processes of the refinery. Among them is the work proposed in [1] for estimating oxygen content in a coke furnace, and the soft-sensor for predicting MAE and SWA acid gases in a sulphur recovery unit or for butane concentration in a debutanizer column [2,3].

Nevertheless, a common issue reported in real industrial applications is that the datasets are generally “data rich but information poor” [4]. Therefore, there is a considerable need to devise intelligent strategies for selecting informative data that extract valuable knowledge. Some researches include preprocessing methods for identifying or even discarding samples that may worsen the model output. For instance, the authors of [5] improve the gasoline dry point prediction accuracy by removing the influence of outliers of the operation data. In the research presented by [6], a Robust Partial Least Square (PLS) method is employed to identify multivariate anomalies, and a Dynamic PLS selects and optimises the input samples that best estimate the naphtha dry-point in an atmospheric vacuum distillation tower. In line with the data selection, authors of [7] include a Gaussian process-based samples selection strategy in order to add informative samples for a dynamical adaptation of the model to present an online adaptive model that infers different propane and ethane quality measurements in the top of a depropanizer column.

Following the same research line, other related works take a step forward by utilising Feature Selection (FS) approaches. In this way, only the relevant input features are selected for creating the model. Authors of [8] include a Genetic Algorithm strategy into their PLS-based soft-sensor to select the most relevant variables for operation and quality control of the ASTM 90% distillation temperature (D90) in a crude distillation unit. Similarly, in order to extract relevant features to estimate the flow rate and the yield of some resultant subproducts (gasoline, diesel oil, coke and LPG) in a Fluid Catalytic Cracking unit, the authors of [9] employ a Recurrent Denoising Auto Encoder and a Cumulative Percent Variance. Another example can be found in [10] where, aiming at selecting the most sensitive features concerning the output value avoiding redundancy problems with correlated features, a double LASSO algorithm integrated into an MLP model is presented to predict the kerosene D95 in a crude distillation unit. Feature selection based on correlation analysis is employed by the authors of [11] for estimating H${}_{2}$S and SO${}_{2}$ acid gases concentration in a Sulphur Recovery Unit and also in the soft-sensor presented in [12] for toluene content estimation.

In the above-mentioned FS approaches, a weight equal to one is assigned to the selected features and zero to the discarded ones. As widely known, a further step is done by the employment of feature weighting (FW) approaches in which a weight between zero and one is assigned in order to represent the degree of relative importance each feature gathers concerning the output label or class. This approach is applied in [13], where the authors include feature weights calculated as the correlation between each feature and the output variable to estimate the butane concentration at the bottom of a debutanizer column.

As observed in the state-of-the-art highlighted above, recent works rely on the context of Industry 4.0, digitising industrial processes [14,15] by proposing soft-sensors that integrate feature preprocessing and ML algorithms. Another hot topic in both industry and academia is automated Machine Learning (autoML) [16,17,18,19,20], which aims at enabling domain experts to build ML applications automatically [21]. As stated in [22], the ideal autoML approach involves data preprocessing, model generation, and model evaluation. Despite data preprocessing being the first task typically in ML approaches, autoML systems have focused on model selection and hyper-parameter searches [23], while data preprocessing still requires considerable human intervention [24]. Aiming at advancing in the automation of learning systems in the context of Industry 4.0, this research presents an autoML approach that searches for the best configuration among well-known normalisation and FW preprocessing techniques as well as among popular ML algorithms in order to create a soft-sensor that complements and supports the operators’ decision-making by classifying the percentage of pentanes in the butane obtained at the end of a debutanizer column, according to the product specifications. The quality of butane is dependent on the percentage of pentanes present in the gas. If the rate exceeds a certain threshold, the product must be reprocessed, and additional costs are incurred. Therefore, several works are devoted to solving this open challenge. Ito et al. [25], based on data obtained from a gas processing plant simulation, infer in an online fashion the concentration of pentanes in a debutanizer column by combining a physical model with heuristic rules. Similarly, the authors of [26] present a NARMAX-based soft-sensor for estimating the pentanes content in butane. The authors utilise data from a real refinery plant where the time tag difference between the input and the output lies in a range of 20–60 min approximately.

In contrast to [25,26], this work aims at predicting 400 min in advance if the percentage of pentanes in butane at the end of the debutanizer column will fulfil the quality standards. Thus, the operators can adjust the process immediately and avoid distilling a product that will not meet the specifications for more than six hours. With that purpose, this work utilises real data from a refinery of the Basque Country, and process variables of the top of the stabilising naphtha towers are employed to create the soft-sensor. The autoML preprocessing phase design and development are based on a novel two-stage methodology that combines normalisation and feature weighting to intelligently transform the input data to reflect each feature’s relative importance for classifying the resulting quality. In order to configure the soft-sensor from the two-stage methodology, in the model generation and evaluation phase, seven well-known classification algorithms are considered [27]: Quadratic Discriminant Analysis (QDA), K-Nearest Neighbours (KNN), Support Vector Classification (SVC), Ridge Regression (RID), Logistic regression (LOG), MultiLayer Perceptron (MLP) and Stochastic Gradient Descent (SGD). Since the purpose of the resulting soft-sensor is to complement the operator decision-making, the model that maximises the classification performance in terms of precision is selected, aiming at maximising the operator’s reliability in the model’s results when performing operational changes in the system.

The remainder of the paper is structured as follows: Section 2 presents an outline of the analysed distilling process. Section 3 describes the methodology proposed for the design and development of the autoML approach that searches for the best configuration of the soft-sensor for class prediction of the percentage of pentanes in butane at the end of the debutanizer unit. Classification results, analysis of the developed soft-sensor and the profit obtained by applying the proposed approach are shown in Section 4. Finally, Section 5 depicts the conclusions and future work.

## 2. Problem Description

**Figure 1:**
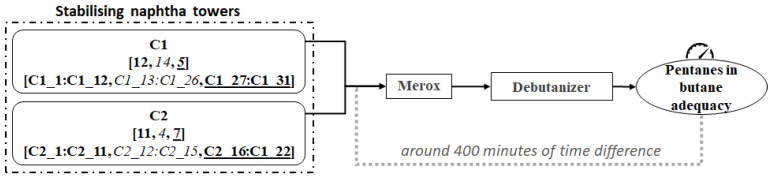
High-level diagram of the analysed process.

In this work, real data from a refinery allocated in the Basque Country are utilised. Figure 1 depicts a high-level diagram of the analysed unit chain, in which crude oil is converted into high-quality gas subproducts.

Columns C1 and C2 in Figure 1 represent two different stabilising naphtha towers. After a refining process of the raw crude, stabilised naphtha and Liquefied Petroleum Gas (LPG) are obtained at the bottom and the top of the columns C1 and C2, respectively. The resulting LPG is then pumped from the top of columns C1 and C2 to Merox, a gas sweetening unit in which the sulphur is removed. Finally, the sweetened gases pass to the debutanizer column, where propane and butane are separated. The estimated duration of the described unit chain, from stabilising naphtha columns to the end of the debutanizer column, is 400 min.

In order to fulfil the specification standards [28], the resultant butane must not exceed a certain threshold of the percentage of pentanes (1.5%). According to the mentioned threshold, the refinery’s interest is to classify the percentage of pentanes in butane as adequate (class 0) or improvable (class 1). Currently, a Dynamic Matrix Control (DMC) control algorithm optimises the distillation process in columns C1 and C2 by adjusting temperature and reflux from the top of the columns according to an estimation of the number of pentanes in the feed. Furthermore, currently, the percentage of pentanes in butane at the end of the debutanizer column is measured online. However, despite the suitability of the DMC, there are some episodes in which pentanes escape from the top of columns C1 and C2. In such scenarios, the deviation from the requirements is detected at the end of the debutanizer column. Then, aiming at estimating the percentage of pentanes in butane 400 min in advance, in this work, the classification is conducted based on the refining process of C1 and C2. With that purpose, from the top of C1 and C2, 31 (C1_1:C1_31) and 22 (C2_1:C2_22) features are collected, respectively. These features at each column gather information about flow, temperature and pressure from operational variables and DMC. The number of features of each column regarding each of these properties are presented in Figure 1 with bold, italic and underlined text, respectively. The process variables information and the pentanes percentage output were recorded every ten minutes for 465 days, from 24 October 2017 to 31 January 2019. Thus, the dataset consists of 66,847 samples described by the 53 features described above.

## 3. Methodology

As mentioned above, this research proposes an autoML approach that selects the best configuration among well-known preprocessing techniques and different ML algorithms. The final objective is to create, based on the process condition in the top of the naphtha stabiliser columns, a reliable soft-sensor that performs the offline model training and the posterior online validation for classifying the percentage of pentanes in butane at the end of the debutanizer column 400 min in advance. This section describes the methodology employed to analyse the dataset and the stages of the autoML approach.

### 3.1. Dataset Evaluation

**Figure 2:**
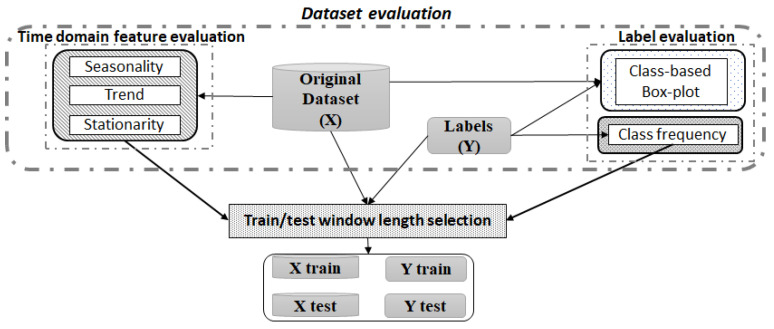
Dataset evaluation diagram.

Figure 2 depicts the procedure proposed for evaluating the dataset and extracting the key information from the data. This section thoughtfully details such procedure and, hence, the mathematical tests proposed for determining the optimal train and test sets for modelling the algorithm. Finally, the analysis of the relationship between the input features and the real labels is detailed, and how the result of such study determines the latter ML algorithms application is explained.

#### 3.1.1. Time Domain Feature Evaluation

In order to extract information from the historical data to predict the future, it is desirable that the data collected over time is representative of current conditions, i.e., it reflects stable equilibrium. This property is called stationary, and this work proposes to check it by means of considering the features of the dataset as time series.

A time series [29,30] is an ordered sequence of observations, and it is defined as a function of three major components: seasonality, trend and random noise. Seasonality and trend are sources of non-stationarity, which means their identification and removal from the time series can result in a clearer relationship between input features X and output Y. In addition, if the random noise component of the time series is not stationary, the statistical properties of the time series evolve over time. In such setting, a shorter sampling interval may be needed to capture key characteristics of the population. With the aim of checking the time series stationarity to select the optimal set (X train) to train the model, seasonality, trends and stationarity of the time series are analysed. The time domain feature evaluation is completed by the analysis of the rolling statistics of the time series.

- Seasonality refers to the repeating variation at regular intervals of time. The data are considered seasonal if a significant autocorrelation [31] coefficient exists at a given lag [32]. The autocorrelation function measures the linear correlation between a time series and a delayed version of itself searching for repeating patterns. Auto-correlation values range between [−1,1], where 1 and −1 values represent total positive or negative correlations between two time series, respectively.
- Trend refers to the general tendency of the features (upward or downward); the Mann–Kendall (MK) test [33] is utilised to ascertain the absence of a trend in the time series. Thus, the null hypothesis $H_{0}$ assumes that there is no trend in the time series and the MK test analyses the sign differences between samples of different moments to discard increasing or decreasing measurements in the time series.
- Stationarity analyses the random development around a constant average of the time series. Augmented Dickey–Fuller (ADF) [34] and Kwiatkowski–Phillips–Schmidt–Shin (KPSS) [35] non-parametric tests were applied with the aim of examining if the time series is stationary and the statistics are consistent over time. The null hypothesis $H_{0}$ of the ADF test states the presence of a unit root, i.e., the series is non-stationary, while the alternative hypothesis assumes the weak stationary. Concretely, ADF states that if a unit root exists, the lagged version of the time series does not provide information for predicting changes in the current value of such time series. In contrast, KPSS test’s $H_{0}$: (1) assumes that the time series is stationary around the trend, and (2) it expresses the time series as the sum of the deterministic trend, random walk and stationary error. Since the possible source of non-stationarity in this expression is the random walk, KPSS checks that the random walk has zero variance, i.e., it does not evolve over time.
- Rolling statistics, such as mean and standard deviation, were also analysed in order to check the stability of the time series over time, as well as to detect changes in the statistical properties of the time series. Thus, if changes are detected with the rolling statistics analysis, the window size selection is conducted considering the frequency of such changes.

#### 3.1.2. Label Evaluation

Considering the real labels Y of the dataset, two analysis are conducted: (1) the frequency of each class occurrence over time is computed in order to select a representative sample population of each class for training (X train, Y train) and testing (X test, Y test) the model, and (2) the discriminative ability of the features can be adverted in the box-plot and hence, the existence/absence of a linear relationship between the input features and the output label will determine the selection of the most appropriate ML algorithm to create the soft-sensor.

### 3.2. Optimal Dataset Split Selection

In order to create a reliable model and to reproduce the offline training and online quality prediction practice, the dataset split is done respecting the temporal order of the data. As determined in Section 3.1.1, if the time series is not stationary, employing all available historical data can disturb the prediction ability of the model due to the evolving time series statistical properties over time. In such scenario, the window length for the training set must be selected in order to capture the variability of the input data and, thus, the current properties of the time series. Likewise, as described in Section 3.1.2, the training set (X train, Y train) is selected in such a way that all classes are represented.

Furthermore, note that in online environments, the moment in which the statistical properties will change with respect to the current condition is not known in advance. Consequently, for the train/test window size selection, a conservative approach is conducted, which considers the possibility of statistical properties drift in the test set.

### 3.3. AutoML Approach Description

**Figure 3:**
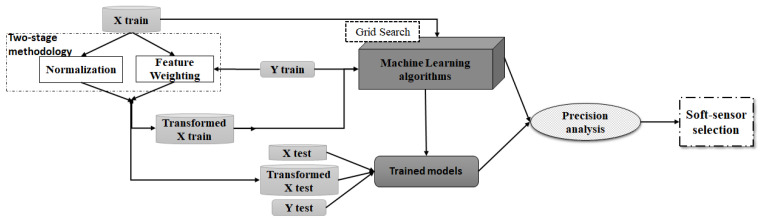
High-level diagram of the proposed autoML approach.

This Section describes all the stages of the autoML approach that selects the best configuration among different well-known preprocessing methods and ML algorithms in order to create the soft-sensor for supporting the decision-making. Figure 3 illustrates a high-level diagram of the proposed autoML approach.

#### 3.3.1. Two-Stage Methodology

The Two-stage methodology is applied in order to transform the original raw dataset $X\subset R^{n\times m}$ into a new transformed one, denoted by ${\tilde{X}}_{FW}^{Norm}\subset R^{n\times m}$, based on normalisation and feature weighting, for representing the relative importance, each feature j={1,…,m} gathers for classifying the samples i={1,…,n} among the different classes. 

(1) Normalisation. It is thought that normalisation equalises the contribution of each feature in the ML algorithm calculations [36]. This is why normalisation methods are commonly applied during the preprocessing step in order to avoid the over-contribution of a set of features due to the magnitudes difference. However, each normalisation method transforms the dataset differently. In addition, each feature is compressed or expanded depending on the normalisation method and its statistical values [37], which ultimately can condition the features’ influence on the ML algorithm calculations and its performance. Since there is no specific normalisation method suitable for all the problems, three of the most commonly employed approaches are selected in this work. All of them are linear transformations based on position and dispersion statistics.

- Standardisation (ST): ${\tilde{X}}_{j}^{ST}=\left(X_{j}-\bar{X_{j}}\right)/\sigma_{j}$∀j∈{1,…,m}. The resultant features are centred around the mean with a standard deviation equal to 1.
- Min–max normalisation (MM): ${\tilde{X}}_{j}^{MM}=\left(X_{j}-\min\left(X_{j}\right)\right)/range\left(X_{j}\right)$, ∀j∈{1,…,m} where $range\left(X_{j}\right)=\max\left(X_{j}\right)-\min\left(X_{j}\right)$. The samples of the resulting features take values between [0,1].
- Median Absolute Deviation normalisation (MAD): ${\tilde{X}}_{j}^{MAD}=\left(X_{j}-\mathrm{Me}\left(X_{j}\right)\right)/\mathrm{MAD}\left(X_{j}\right)$, ∀j∈{1,…,m}. In contrast to the other techniques, MAD normalisation is considered a robust transformation [38] as the calculation of the median Me is not affected by the presence of outliers.

Since each normalisation method (ST, MM and MAD) depends on different statistics, and given that each feature is transformed differently depending on its statistical characteristics, it is expected that a different subset of features predominate for each normalised dataset (${\tilde{X}}^{ST},{\tilde{X}}^{MM}$ and ${\tilde{X}}^{MAD}$). Then, in this work, the range of the features is employed as an indicator of the influence of each feature in the algorithm performance [39]. To facilitate the comparison between features of the same dataset, the ranges are divided by the maximum range of the dataset $range\left({\tilde{X}}_{j}^{Norm}\right)/max\left(\left\{range\left({\tilde{X}}_{j}^{Norm}\right)|j=\left\{1\ldots ,m\right\}\right\}\right)$. This way, the most influencing features, i.e., with a range close to the maximum one, present a normalised range close to 1. In contrast, features that present range value much lower than the maximum one, being, in comparison, insignificant ranges, will present a normalised range close to 0. 

(2) Feature Weighting. Feature weighting methods transform the features of the dataset to be representative of the relative information each gathers for estimating the output. This transformation is conducted by a vector **w** of feature weights, where the components represent the relative importance of each feature. Note that each weight has a value in the range from 0 to 1, so the sum of $w_{j}$ for all j={1,…,m} is 1. Among the FW methods, the filter approaches calculate the feature weights independently from the ML algorithm. If information on the labels is employed for computing the weights, the FW approach is considered supervised. Three supervised filter FW methods are applied in this research: two well-known methods, Random Forest and Mutual Information, both based on Information Theory and the Adapted Pearson correlation [37], a statistical-based method previously proposed by these authors. Random Forest calculates the feature weights conjointly, while Mutual Information and Adapted Pearson correlation estimate the weights for each feature in an independent manner, i.e., without considering the remaining ones. The three FW methods are briefly described below:

- Adapted Pearson correlation (P): this statistical-based FW method is an adaptation of the Pearson correlation coefficient for handling categorical and continuous features. It aims at estimating the relative importance of each feature for separating the classes in classification problems. With that purpose, the proposal presented in [37] utilises the labels of the dataset to separate the samples according to the class. Thus, the labels are encoded as the centroid of the samples that correspond to such label. Then, for each component of the vector of weights, the absolute value of the Pearson correlation coefficient is estimated between each feature and the corresponding component of the encoded label. Finally, the weights vector is divided by the sum of their components to obtain the vector of weights $w^{P}$, so $\sum_{j=1}^{m}w_{j}^{P}=1$.
- Random Forest classifier (RF): Random Forest [40] is a decision tree-based ensemble-learning ML algorithm utilised for different tasks, such as classification or regression problems. In addition, it is also widely employed for calculating the relevance of the features for estimating the output, according to their contribution in the trees employed for creating the forest. Each tree in the ensemble employs bootstrapping, which, together with an elevated number of trees and the tree splitting strategy, are randomness sources that decrease the variance of the estimations. Thus, in this work, the RF employed as the FW method is constructed by 100 decision trees. The final feature weight vector $w^{RF}$ is calculated as the mean of the features importance of 30 RF-based models. Thus, in total, 3000 decision trees are considered. Each decision tree is constructed from a random subset of features of length equal to the square root of the total number of features of the dataset. A leaf node requires a minimum of one sample, while all the nodes with more than one sample are considered internal nodes. The sub-sample set employed for training each tree presents the same size as the original dataset, but, with bootstrap, this set is drawn with replacement. Once the algorithm is trained, the relative importance of each feature is calculated by the Mean Decrease Gini [41], which computes the mean of the weighted impurity decrease of all the nodes of all the trees where the feature is used. In this work, the scikit-learn package [42] of python has been used for the estimation of $w^{RF}$.
- Mutual Information (MI): this FW method measures the degree of mutual relatedness between a feature and the labels, which can be interpreted as the amount of shared information between them. MI employs joint and marginal probability distributions to compute the calculations, which are generally unknown in real problems. Again, the scikit-learn package of python is utilised [42], which adds a small amount of noise to continuous variables in order to remove repeated values, and employs a nearest neighbour method [43,44] for estimating the MI. In this work, the number of neighbours *k* is set to 3, since small *k* reduces systematic errors [43,44]. For each feature, the weight ranges from 0 to 1, and, the higher the values, the higher the relationship between the feature and the labels. In order to be the sum of the components of the vector of weights $w^{MI}$ equal to 1, the estimated weights are divided by the sum of the feature weights.

The feature weights $w^{P},w^{RF}$ and $w^{MI}$ along with the normalisation approaches above-described are employed for creating the transformed dataset ${\tilde{X}}_{FW}^{Norm}$, as it will be explained next. 

(3) Transformed Dataset Calculation. As depicted in Figure 3, the two-stage methodology lies in combining both normalisation and feature weighting to intelligently transform the raw dataset. By this means, normalisation acts over the magnitude differences among the features in order to extol the resulting importance of the representativity of the features.

Then, the two-stage based transformation results from multiplying each normalised feature by the corresponding feature weight, ${\left({\tilde{X}}_{P}^{ST}\right)}_{j}=w_{j}^{P}\cdot {\left({\tilde{X}}^{ST}\right)}_{j}$, ..., ${\left({\tilde{X}}_{MI}^{MAD}\right)}_{j}=w_{j}^{\mathrm{MI}}\cdot {\left({\tilde{X}}^{MAD}\right)}_{j}$, respectively, for j∈{1,…,m}.

This transformation can be expressed in matrix notation as follows: given the normalised dataset ${\tilde{X}}^{Norm}$ and the diagonal matrix $diag(w_{1}^{*},\ldots ,w_{m}^{*})$ formed by the elements of the vector of weights $w^{*}$, the transformed dataset is calculated as,

(1) $${\tilde{X}}_{FW}^{Norm}={\tilde{X}}^{Norm}\cdot diag\left(w_{1}^{*},\cdots ,w_{m}^{*}\right)=\left(\begin{array}{cccc} \tilde{x_{11}} & \tilde{x_{12}} & \cdots & \tilde{x_{1m}}\\ \tilde{x_{21}} & \tilde{x_{22}} & \cdots & \tilde{x_{2m}}\\ \vdots & \vdots & \vdots & \vdots \\ \tilde{x_{n1}} & \tilde{x_{n2}} & \cdots & \tilde{x_{nm}} \end{array}\right)\cdot \left(\begin{array}{cccc} w_{1}^{*} & 0 & \cdots & 0\\ 0 & w_{2}^{*} & \cdots & 0\\ \vdots & \vdots & \ddots & \vdots \\ 0 & 0 & \ldots & w_{m}^{*}. \end{array}\right)$$

The matrix resultant from Equation (1) contains in each column the normalised weighted feature. Thus, in this work, from the combination of each of the three selected normalisation methods (ST, MM, MAD) represented by ${\tilde{X}}^{Norm}$ in Equation (1), with each of the feature weights vectors $w^{*}\in \left\{w^{P},w^{RF},w^{MI}\right\}$ generated by the three FW approaches (P, RF, MI), a total of nine transformed datasets are obtained: ${\tilde{X}}_{P}^{ST}$, ${\tilde{X}}_{P}^{MM}$, ${\tilde{X}}_{P}^{MAD}$,${\tilde{X}}_{RF}^{ST}$, ${\tilde{X}}_{RF}^{MM}$, ${\tilde{X}}_{RF}^{MAD}$, ${\tilde{X}}_{MI}^{ST}$, ${\tilde{X}}_{MI}^{MM}$ and ${\tilde{X}}_{MI}^{MAD}$.

#### 3.3.2. Machine Learning Algorithms

Once the original data have been intelligently transformed by Equation (1) and the datasets with features representative of their relative importance for discriminating the class labels have been obtained, different ML classification algorithms are applied.

Specifically, seven ML classification algorithms [27] from scikit-learn [42] are employed: Quadratic Discriminant Analysis (QDA), K-Nearest Neighbours (KNN), Support Vector Classification (SVC), Ridge Regression (RID), Logistic Regression (LOG), MultiLayer Perceptron (MLP) with one hidden layer and Stochastic Gradient Descent (SGD).

QDA utilises a quadratic decision surface to separate the classes assuming that each class density function follows a multivariate Gaussian distribution. It calculates different covariance matrices for each class, which are regularised by the hyper-parameter reg_param. The algorithm KNN classifies each sample based on the class membership of its *k* neighbours, i.e., the *k* closest samples measured in terms of Euclidean distance. In contrast, SVC creates a hyper-plane, allocated between the supporting vectors, for separating the samples of both classes. It includes a soft-margin hyper-parameter *C* for controlling the misclassification cost. In addition, the SVC relies on the kernel trick, which allows operating in a higher dimension through inner product between pairs of data, and its hyper-parameter γ regulates the influence of samples selected by the model as support vectors. The RID algorithm is a regularised version of the Ordinary Least Squares regression model, where α is a regularisation hyper-parameter for controlling the regression parameters. In the case of the LOG algorithm, it employs the logistic function to classify the samples, and like SVC, it includes a hyper-parameter *C*. The MLP employed in this work is a feedforward artificial neural network with a hidden layer composed of a user-defined number of neurons. Each neuron applies an activation function to a weighted linear summation of the input values, and the final output is a weighted sum. Finally, SGD is an optimisation algorithm for minimising a loss function implemented to regularise linear models, where the hyper-parameter α controls the strength of the regularisation.

Once the employed algorithms and their hyper-parameters have been described, a grid search (GS) algorithm is employed to select the hyper-parameters that maximise the score in terms of the selected performance metric described in Section 3.3.3. Table 1 collects the hyper-parameters employed in the GS for each ML algorithm and the total number of possible combinations.

Some of the selected algorithms, i.e., MLP and SGD, are stochastic and depend on the initialisation. Hence, 10 random initialisations are launched per combination of hyper-parameters, and the mean performance measure value is calculated. Then, the hyper-parameters with highest mean value are selected for the ML algorithm configuration. Similarly, once the optimal hyper-parameters have been selected, the results for the ML algorithms are given by the maximum, mean, standard deviation and minimum performance values from 30 random initialisations.

In order to validate the suitability of the two-stage methodology, the classification results of the nine transformed datasets resulting from the two-stage methodology (${\tilde{X}}_{P}^{ST}$, ${\tilde{X}}_{P}^{MM}$, ${\tilde{X}}_{P}^{MAD}$,${\tilde{X}}_{RF}^{ST}$, ${\tilde{X}}_{RF}^{MM}$, ${\tilde{X}}_{RF}^{MAD}$, ${\tilde{X}}_{MI}^{ST}$, ${\tilde{X}}_{MI}^{MM}$ and ${\tilde{X}}_{MI}^{MAD}$) are compared with those from the original and the normalised datasets ($X,{\tilde{X}}^{ST}$, ${\tilde{X}}^{MM}$ and ${\tilde{X}}^{MAD}$).

#### 3.3.3. Precision Analysis

After the application of the ML algorithms described in Section 3.3.2 to the nine resultant datasets from the two-stage methodology and to the raw and the normalised ones, the performance of the algorithms over each dataset is evaluated for comparison purposes. The classification ability of each model can be visualised through the confusion matrix; it reflects the relationship between the predicted classes and the real ones. Thus, in the diagonal, the number of samples correctly predicted as class 0 or 1 are collected, which are also known as true negative (TN) and true positive (TP), respectively. In contrast, the elements out of the diagonal represent the samples wrongly classified. More concretely, the cell (0,1) collects the number of samples classified as 1 with 0 their real label, known as false positive (FP) samples; and the cell (1,0) presents the false negative (FN) cases, those erroneously classified as 0 when they really belong to the class 1. The sum of the elements of the confusion matrix TP+TF+FP+FN=N is the total number of classified samples.

From the elements of the confusion matrix, different metrics are utilised for performance evaluation. A commonly employed performance metric is the accuracy (TP+TN)/N, defined as the proportion of samples correctly classified. However, it is not recommended for imbalanced datasets, since a high overall accuracy can be reached by compromising the minority class. Thus, there are other metrics especially designed for measuring the classification performance in terms of class 1, such as precision =TP/(TP+FP) and recall =TP/(TP+FN). Precision measures the proportion of samples the model predicts with label 1 that really correspond to such class. Thus, the higher the precision value, the lower the number of samples belonging to class 0 the are misclassified as 1. In contrast, recall represents the proportion of samples of class 1 detected by the model. Then, a low recall value corresponds to a model with poor ability for recognising the samples of class 1.

The main interest of the refinery is to complement the operators decision making with a highly-reliable predictor that detects when an improvable quality subproduct (class 1) is resulting from the process, with the minimum false alarms, so high-cost operational changes are avoided. Then, for the automatic soft-sensor creation, in the autoML approach, the precision is selected as the principal performance measure.

## 4. Results

In this section, the results obtained from the inspection of the dataset and the application of the methodology described in Section 3 are collected. Thereafter, an analysis of the profit the refinery would obtain from the application of the developed soft-sensor is presented.

### 4.1. Dataset Evaluation

An analysis of the dataset was conducted based on points remarked in Section 3.1.

#### 4.1.1. Time Domain Feature Evaluation

**Figure 4:**
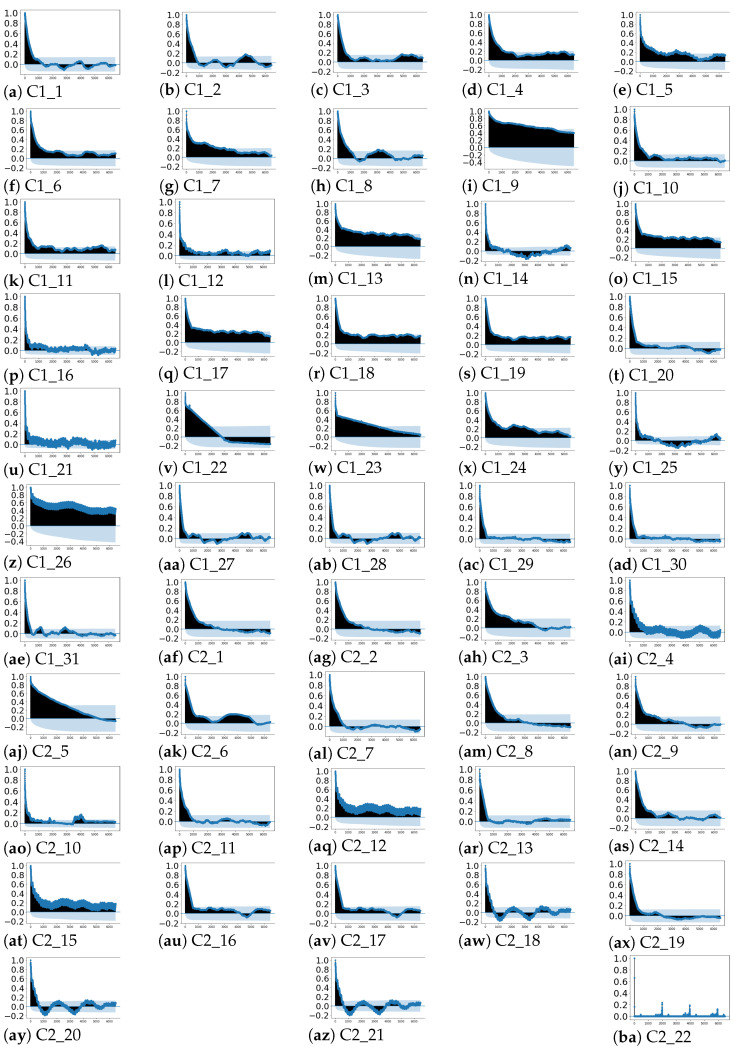
Auto-correlation plots of each feature of the dataset with lags up to 45 days.

The obtained properties that characterise the temporal behaviour of the features are analysed below:

- Seasonality: Figure 4 depicts the auto-correlation of each feature with respect to itself considering a maximum lag of 6480 samples (45 days, determined by expert knowledge). As it can be observed in Figure 4a–ba, the auto-correlation values decrease with the lag increment. In most (50 out of 53) of the time series, the auto-correlation coefficient rapidly decreases to values lower than 0.4, except for Figure 4i,z,aj. In the latter cases, the auto-correlation coefficients decrease slowly with the lag increase, but the values are lower than 0.8. Then, from the auto-correlation plots no seasonality is observed.
- Trend: the *p*-values obtained from applying the non-parametric Mann–Kendall test are shown in Table 2. It can be observed that 17 time series (C1_1:C1_4,C1_6:C1_9,C1_11, C1_17, C1_24, C2_3, C2_9:C2_10,C2_13:C2_14) present *p*-values lower than 0.05; for those features, $H_{0}$ can be rejected. In the rest of the cases, there is no evidence for rejecting the hypothesis of no tendency. Thus, in 36 out of 53 time series, no trend is observed.
- Stationarity: stationarity is checked through the non-parametric ADF and KPSS tests, respectively. The obtained *p*-values for the ADF test are shown in Table 3. In 30 out of the 53 time series, the *p*-values marked with italic text in Table 3 range between [0.05,0.488], so in these cases (and considering a significance level of 0.05) the null hypothesis can not be rejected and, consequently, they are non-stationary. Accordingly, $H_{0}$ can be rejected for the rest of the cases that have obtained *p*-values between [0,0.048].
  Moreover, the *p*-values resulting from the KPSS test are depicted in Table 4.

Based upon the significance level of 0.05, there is evidence for rejecting $H_{0}$, and hence defining as non-stationary 51 out of the 53 time series—marked with bold text in Table 4. C1_14 and C2_2 are the only ones with *p*-value = 0.1>0.05.

All in all, it can be concluded from the *p*-values collected in Table 3 and Table 4 that the time series are non-stationary, and from Figure 4 and Table 2 and Table 4 that such non-stationarity is not caused by seasonal or tendency components.

**Figure 5:**
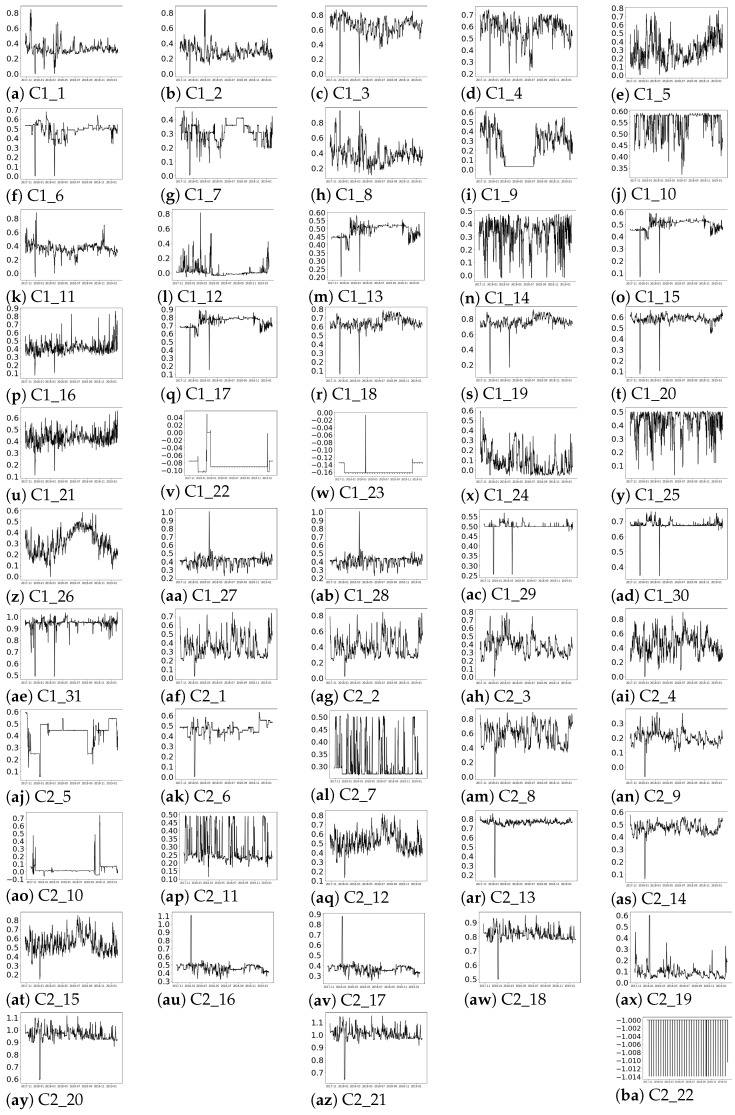
Rolling mean with a window of size 24.

**Figure 6:**
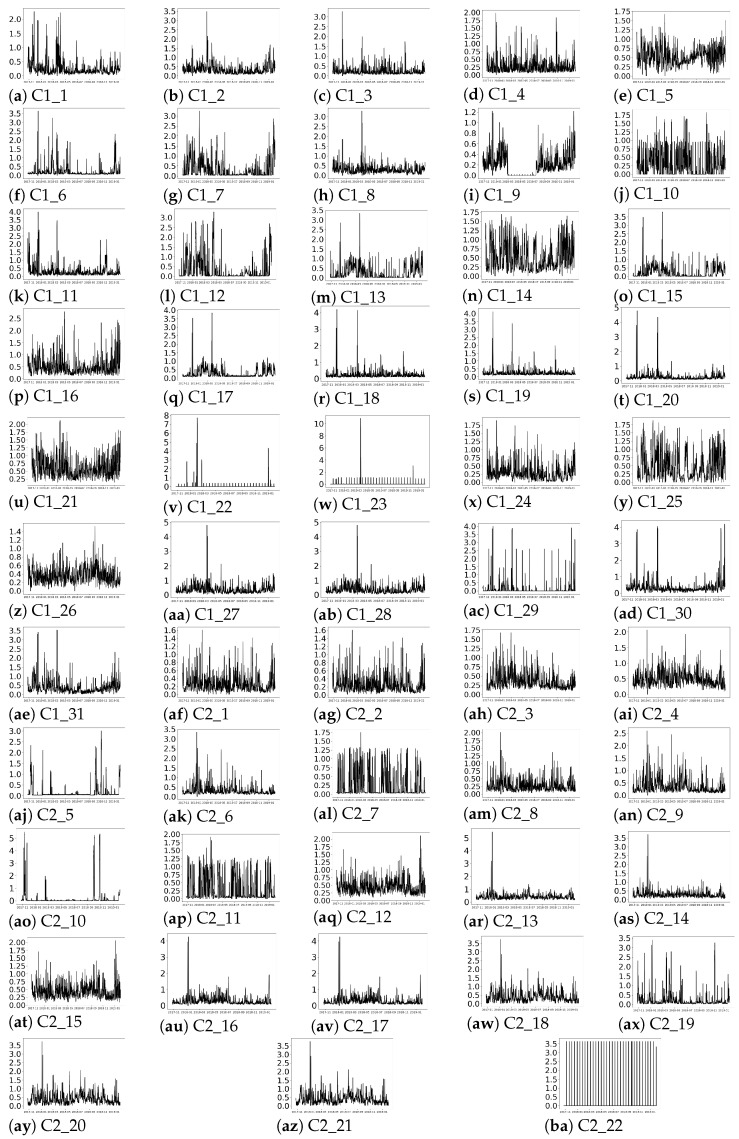
Rolling standard deviation with a window of size 24.

- Rolling statistics: the evolving behaviour of the series over time is depicted in Figure 5 and Figure 6 where rolling mean and standard deviation are estimated, based on expert recommendation, with a window of length 24, i.e, 4 h. (Aiming at preserving the confidentiality of the data, Figure 5 and Figure 6 have been scaled.)

Figure 5a,ba reflect that no seasonality—in terms of repeating patterns—are observed over time, which is in accordance with the results shown in the auto-correlation plots in Figure 4. In terms of trend, as concluded from values depicted in Table 2, there was no evidence for rejecting the tendency test except for 17 time series. In the presence of trend, the mean values of the time series would decrease or increase with time. However, in Figure 5, no stable decay or increase in the mean value is observed, except in Figure 5e,z for the period of September 2018 to 2019. Therefore, despite in Table 2, the trend hypothesis can not be rejected according to the MK test for 36 of the time series as the rolling mean does not show such trend in 34 out of those 36 time series.

Finally, Figure 6 collects the rolling standard deviation of the time series. As explained in Section 3.1, a stationary time series is developed around a constant point over time, presenting stable statistics, i.e., constant mean and standard deviation values over the time series arise. However, in Figure 6a,ba, it is observed that the rolling standard deviation presents significant peaks over time. In cases like Figure 6v,w, most of the peaks are of similar height and they appear at almost constant periods of time, but in the rest of the time series, the peaks in the rolling standard deviation are not so uniform. The variations detected over time in the rolling standard deviations values from Figure 6 reinforce the conclusions about the non-stationarity of the time series obtained with ADF and KPSS tests. Furthermore, in Figure 6c,q,r,s,ac,aj,ao, it can be observed that the values with the highest standard deviation values are found with a varying time separation of 1.5 to 4 months.

Therefore, considering (1) the results in Table 3 and Table 4 regarding the non-stationarity of the time series, (2) the aforementioned non-uniformity of the rolling standard deviation along the time series, (3) expert knowledge advice, and (4) the conservative strategy, the conclusion obtained is that the window for selecting the optimal train and test set must be 3 months.

#### 4.1.2. Label Evaluation

**Figure 7:**
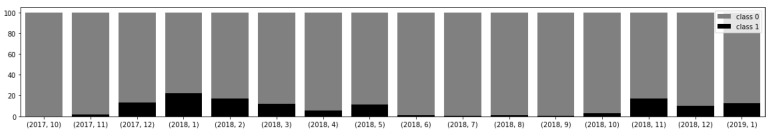
Percentage of samples belonging to each class by month in the recorded time.

Regarding the class samples distribution, the analysis determines that the dataset is highly imbalanced as just 15% of the samples belong to class 1. In addition, the distribution of the classes varies over time, as it can be observed in Figure 7, where up to four consecutive months with less than 1.255% of samples belonging to class 1 are found. Consequently, such periods do not fulfil the class distribution required for training the model; the samples selected for both training and testing must be representative of both classes. As stated in Section 4.1.1, the optimal window comprises 3 consecutive months of historical data. Consequently, the train/test set are obtained from consecutive periods, where approximately 15% of the samples belong to class 1.

**Figure 8:**
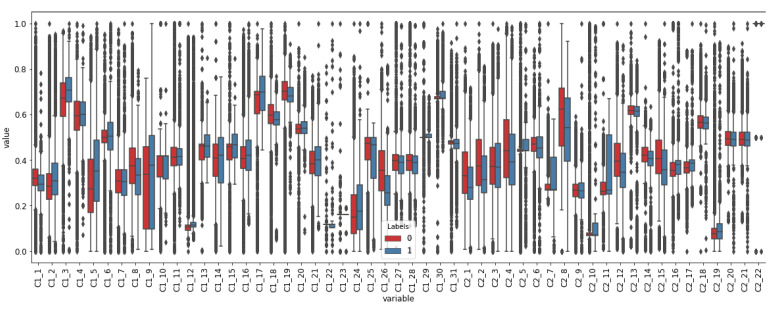
Box-plot of the features distinguishing between class membership of the samples.

Conversely, Figure 8 depicts a class-based box-plot. The bottom of the lower whisker and top of the upper whisker represent the minimum and maximum values, respectively and the points above or below the whisker are outliers. The top, medium and bottom of the box depict 75, 50 and 25 percentiles (Q3, Q2 and Q1), respectively. In Figure 8, it can be observed that, for each feature, if comparing the interquartile range, the box representing the samples of class 0 overlaps with the box of class 1. Q1 and Q3 estimated for class 0, and the values of class 1 are the same in features C1_22, C1_23 and C2_22. The box representing class 1 is totally contained in the values that the box representing class 0 takes in features C1_4 and C1_11. Similarly, in C1_6, C1_9, C1_10, C1_14, C1_16, C1_21, C1_25, C1_29, C1_31, C2_3, C2_6, C2_7, C2_9, C2_10, C2_19, the box representing class 0 is totally overlapped with the box of class 1. Thus, the overlapping level between the samples from Q1 to Q3 from one class with respect to the other class is up to 100%. The lowest overlapping proportion of the interquartile range of class 1 contained in the interquartile range of class 0, 12.095%, is found in C2_5, but in this feature, the 69.494% of the interquartile range of class 0 overlaps the interquartile range of class 1. Then, the classes are not linearly separable in any of the features. Based on these results, ML algorithms that handle the non-linearity of the features are considered in the autoML approach for creating the soft-sensor.

### 4.2. Optimal Dataset Split

As explained in Section 3.1, if the time series are not stationary, the employment of the whole historical data can disturb the ability of the model for predicting the next time steps. Thus, a chronologically ordered subset of samples from the dataset is selected for modelling the problem. Then, based on (1) the results obtained in Section 4.1.1 about the evolution of the statistical properties of the time series over time, and (2) the results in Section 4.1.2 about the class distribution over time, the conservative period from 17 December 2017 to 15 March 2018 is selected. For the offline model training, the first two months are employed as X train and Y train, while the following one is utilised for the posterior online validation (X test, Y test).

### 4.3. AutoML Approach Results

In the following, the results of the application of the two-stage methodology described in Section 3.3.1 are presented.

#### 4.3.1. Normalisation

**Figure 9:**
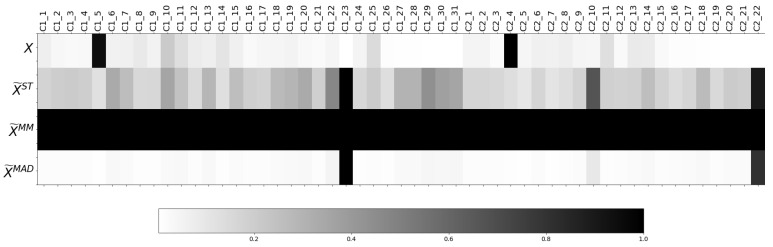
Normalised ranges of the features of the raw and the normalised dataset ($X,{\tilde{X}}^{ST},{\tilde{X}}^{MM}$ and ${\tilde{X}}^{MAD}$).

As described in Section 3.3.1, three different normalisation methods are employed in this work. Aiming at comparing the effect of each normalisation method, the normalised ranges of training sets of the raw *X* and the normalised datasets ${\tilde{X}}^{ST}$, ${\tilde{X}}^{MM}$ and ${\tilde{X}}^{MAD}$, respectively, are depicted in each row of Figure 9.

As Figure 9 shows, the dominating feature for each dataset is different depending on the normalisation method employed, and they do not match with those from the raw dataset *X*. The two dominant features in ${\tilde{X}}^{ST}$ and ${\tilde{X}}^{MAD}$ are C1_23 and C2_22, but in the ${\tilde{X}}^{ST}$ dataset, other features also take values higher than 0.4, while, in ${\tilde{X}}^{MAD}$, the contribution of the remaining features, in terms of range, are insignificant in comparison with C1_23 or C2_22. Therefore, from Figure 9, it is observed that the features’ dominance varies depending on the selected normalisation method. That being so, the normalisation method selection affects the features’ contribution and, therefore, conditions the ML algorithm performance.

#### 4.3.2. Feature Weighting

**Figure 10:**
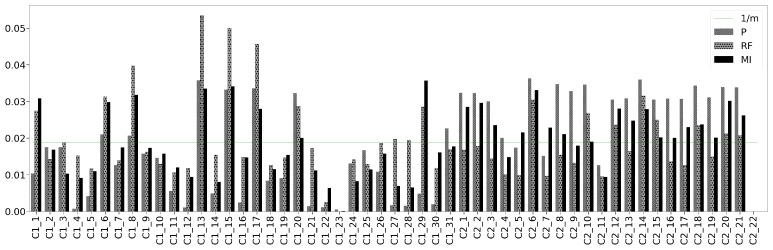
Feature weights estimated by each feature weighting method.

Figure 10 depicts the weights $w^{P},w^{RF}$ and $w^{MI}$ estimated for each feature with respect to the label output by the three FW methods P, RF and MI, respectively, as described in Section 3.3.1.

The horizontal line in Figure 10 refers to the weight each feature would have if all of them presented the same relative importance (1/m) for estimating the output. In contrast, it is observed that the relative importance value assigned to each feature by each FW method varies. The standard deviation measures, with respect the mean, the general deviation of the weights assigned by a FW method. In the case of equal weights, the standard deviation of the weight values is zero. However, the standard deviation of the proportional weight values—the estimated weights divided by the maximum one’s value—estimated for each FW method is higher than 0.2. Thus, from the dispersion of each FW method and Figure 10, it is observed that each feature is assigned a different weight value. In addition, as Figure 10 depicts, for a given feature, the computed feature weights vary depending on the FW method employed for their calculation, such as in feature C2_17, where the weight assigned by RF is 55.734% of the weight value assigned by P.

Table 5 collects the most relevant features according to each FW method, whereas Table 6 gathers the features with weight values lower than 1/m for any FW method. Note that in Figure 10, the weight value of the most influencing features estimated by P and MI FW methods are closely followed by the weight values of other features. In contrast, the weights estimated by RF present a higher difference between the most influencing features and the rest.

This work proposes to quantitatively measure the distribution of the weights as the difference between the maximum weight calculated with respect to the mean of the weights $max\left(w_{j}^{*}\right)-\bar{w^{*}}$. In addition, the cumulative absolute difference (CAD) $\sum_{j=1}^{m}\left|w_{j}^{*}-1/m\right|$ between the weights and the ideal weight (1/m) is presented as a measure of the discriminant power of each FW method.

In the first row of Table 7, RF presents the highest difference between the central tendency weights value and the maximum one. In contrast, the obtained values for P and MI are similar. In terms of CAD, it is observed that the weights obtained with the Pearson FW method are those that most differ from the ideal ones (1/m). Therefore, according to this method for the analysed dataset, the Pearson FW method is the most discriminant one for assigning weights to the features.

#### 4.3.3. Two-Stage Methodology

Figure 9 shows in each row the normalised ranges of the training sets of the resulting transformed datasets from the two-stage methodology described in Section 3.3.1.

**Figure 11:**
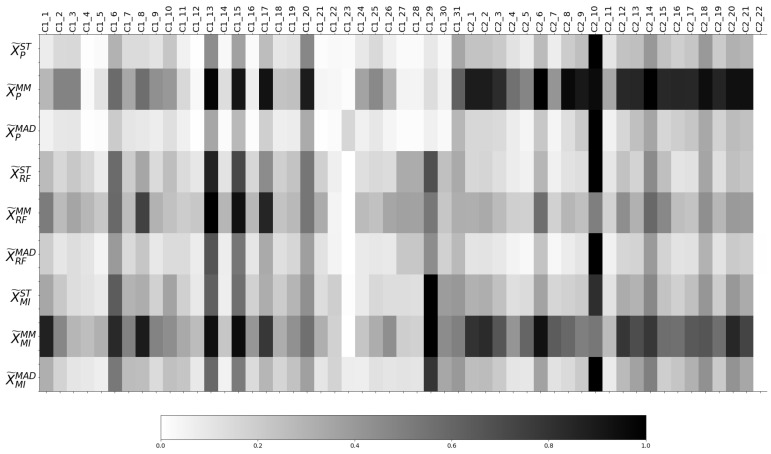
Normalised ranges of the features of the resulting dataset after applying the proposed two-stage methodology.

The proposed two-stage methodology states that the influence of the features for each transformed dataset is computed from the combination of each normalisation method with the weights estimated by each FW method. Such influence can be observed in Figure 11, and it is clearly proven that it varies considerably for each transformed dataset as both the normalisation method and the weight estimation clearly determines the dataset transformation.

Regarding the application of the weights $w^{P}$ estimated with Pearson, similarly to Figure 10 where the features of column C2 present higher values than C1, in the three first rows of Figure 11, the normalised ranges of various features regarding C1 (C1_4, C1_12, C1_21, C1_22, C1_27, C1_28, C1_30) are close to zero. In contrast, the cells corresponding to the transformation conducted by RF and MI methods show that, except for C1_23, the features of C1 present a range value of at least 10% of the value of the maximum feature of the dataset.

Furthermore, in Figure 10, the weights estimated by the three FW methods for C2_22 and C1_23 are approximately zero. In Figure 11, C2_22 presents a normalised range of zero, while, due to the high normalised range ${\tilde{X}}^{MAD}$ in C1_23, this feature presents a higher or equal normalised range than features that presented higher weight in Figure 10, such as C1_27, C1_28 or C2_11. Similarly, recall that in Table 5, the most important features according to each FW method are collected. However, in Figure 9, it can be observed that, after the joint employment of weights estimated with P or RF and ST or MAD normalisation methods, or MI combined with MAD normalisation, the feature with the highest contribution in terms of the range is C2_10—which does not appear in Table 5—but in Figure 9, it represents the third highest value in terms of ranges in ST and MAD. Thus, it can be concluded that the FW weights can be significantly disturbed by the normalisation methods.

#### 4.3.4. Machine Learning Algorithms and Performance Analysis

Once the transformed datasets have been obtained through the proposed two-stage methodology, the ML algorithm is applied, as described in Section 3.3.2. The precision results obtained over the entire month that comprise the test sets by each ML algorithm with the optimal hyper-parameters selected by the GS are collected in Table 8.

As described throughout this paper, each normalisation method transforms a given dataset differently. In addition, the application of weights calculated by a particular FW method is affected by the normalisation factors employed to normalise the dataset. Then, in order to experimentally validate it, Table 8 collects the precision reached by different ML algorithms from the raw and the normalised datasets as well as from the application of the two-stage methodology. As depicted in Table 8 and remarked with bold text, the proposed two-stage methodology outperforms, in every selected ML algorithm, the obtained results by the raw and normalised datasets. For example, in QDA the precision increases from 64.286% to 90% and 100% with ${\tilde{X}}_{MI}^{ST}$ and ${\tilde{X}}_{MI}^{MAD}$, respectively. In the case of MLP, from a mean precision value of 68.631% in ${\tilde{X}}^{MM}$, the two-stage methodology obtains mean precision values higher than 71% for all the combinations, reaching 95.180% of the mean precision value with the ${\tilde{X}}_{RF}^{MAD}$ dataset. Regarding the results obtained from applying the two-stage methodology by different FW methods, the RF method obtains the best precision results for KNN, SVC, RID, LOG and MLP algorithms. These obtained values are closely followed by the reached ones with the P FW method in KNN, RID and LOG ML algorithms. In contrast, the P FW method reaches the maximum precision value only for the SGD ML algorithm, and the MI FW method uniquely outperforms in the QDA ML algorithm compared to the results obtained by the other two FW methods. As described in Section 3.3.1, RF is the only FW method included in this work that considers all the features conjointly, while P and MI independently calculate each feature’s relative importance. Furthermore, note that the FW methods that obtain better and worst results for this problem are RF and MI, respectively, being both information theory-based methods. In contrast, the statistical-based method P reaches similar precision values to RF. That being so, due to the intrinsic characteristics of the FW methods formulation, P, RF and MI are considered the most suitable ones to include in the autoML approach.

The autoML approach presented in this paper selects the best configuration among different well-known normalisation and FW preprocessing methods and various commonly used ML algorithms to create a reliable soft-sensor in terms of precision. More concretely, the models with precision values higher than 95% are preselected for further analysis. Thus, QDA with ${\tilde{X}}_{MI}^{MAD}$, RID with ${\tilde{X}}_{P}^{MM}$, ${\tilde{X}}_{RF}^{MM}$ and ${\tilde{X}}_{MI}^{MM}$ datasets, and LOG with ${\tilde{X}}_{P}^{ST}$ and ${\tilde{X}}_{RF}^{MM}$ datasets are chosen as possible soft-sensors. Table 9 collects the percentage of precision and recall reached by each selected model for different time horizons from the month that comprises the test set.

Table 9 shows that, for the different time horizons, the preselected approaches reliably predict the samples that do not fulfil the specification requirements. Hence, these approaches provide a high degree of confidence to the operator when changing the process operation. However, from the second week of test sets, Table 9 displays a significant decay in the percentage of recall estimated by the preselected approaches. In fact, after two weeks, they all start failing to detect more than 85% of the samples of class 1. The time series non-stationarity stated in Section 4.1 justifies the performance loss along the time and the need of adaptive methods that retrain the model with respect to the drifts in the process. Then, despite the conservative strategy described in Section 4.2 for the train/test set window length selection of a maximum of three months, the following analyses focus on the first two weeks of the test set before the drift.

**Figure 12:**
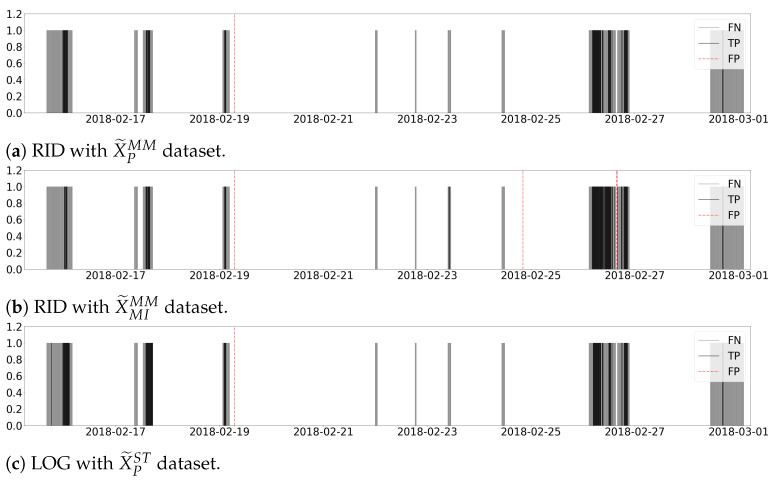
Classification results of the selected models.

Figure 12 depicts the graphical representation of the models with recall higher than 20% from Table 9. The grey vertical lines represent the False Negative (FN) samples, and the black vertical lines represent the True Positives (TP) samples. Finally, the vertical red dash-dotted lines, with a length of 1.2, are the False Positive (FP) samples that the soft-sensor expects to minimise. Thus, Figure 12 displays the reliability of the model for correctly classifying samples from class 1, in spite of not being able to detect all the improvable samples.

The refinery’s main interest is to complement the operators’ decision-making with a soft-sensor that reliably detects samples of class 1 to adjust the process if necessary, minimising the operational changes when the refinery is correctly processing samples that fulfil the specifications requirements. Then, from Table 9 and Figure 12, the model resulting from the LOG ML algorithm over the ${\tilde{X}}_{P}^{ST}$ transformed dataset is selected for creating the soft-sensor as it has a good trade-off between precision and recall.

Notice that the soft-sensor estimates a new virtual measurement every 10 min given the dynamics of the change of the percentage of pentanes from adequate (class 0) to improvable (class 1). However, once the percentage of pentanes transits to class 1, from the domain expert’s perspective, the required operational changes would be applied (1) under an improvable regime persisting during a significant period of time, as next detailed in Section 4.4; or (2) under the operator’s consideration based on the operational variables information analysis after the first alarm from the soft-sensor. This is consistent with the decision of using the precision metric in the training process of the proposed autoML approach.

In Figure 12, the resultant subproduct that does not meet the constraints (class 1) regime in 15 February 2018 persists for 11 h and 50 min. In this case, the selected soft-sensor creates the first alarm 130 min after the first improvable level occurs, which results in an improvement of 270 min with respect to the systems that currently operate in the refinery. Similarly, on 17, 19 and 26 February 2018 it takes only 80, 40 and 120 min, respectively, for the mentioned soft-sensor to detect the subproduct quality deviation. Then, given the high reliability of the presented soft-sensor, the operator can confidently apply high-cost operational changes in order to reduce the disturbances due to an improvable percentage of pentanes.

### 4.4. Profit per Hour Provided by the Soft-Sensor

The logistic regression algorithm applied to the data set transformed by the two-stage methodology based on ST and P is selected for the soft sensor. Although the recall obtained for a two-week time horizon is only 25.485%, the precision is 98.925%. Therefore, the soft sensor is highly reliable in reporting that a sample does not meet the specification requirements. The soft-sensor is created based on the operational information from the top of columns C1 and C2, as depicted in Figure 1, recorded 400 min before the refining process ends. Thus, the operators can early react early by adjusting the process at Merox or at the debutanizer column in order to recover the resultant subproduct quality.

The refineries operate with a high quantity of material. Thereupon, even a deviation of the requirements for a short time involves a high impact on the refinery profit. Next, the economic profit derived from the soft-sensor detection of butane that does not fulfil the specification requirements is calculated.

**Figure 13:**
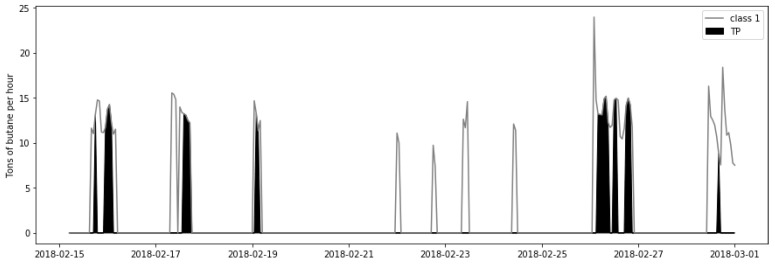
Tons of butane per hour that do not fulfil the specification requirements.

The grey line in Figure 13 depicts the total amount of butane per hour resulting from the distilling process described in Section 3 that does not fulfil the specification requirements. The black area of Figure 13 represents the amount of butane that does not meet the specifications correctly detected by the soft-sensor. The quantity of butane resulting from the distilling process is calculated based on data from the refinery. For the units conversion, from the m${}^{3}$/L of butane flow measured at the end of the unit chain to the tons of butane (Figure 13) utilised to calculate the final profit, a product density value equal to 0.575 kg/L is employed according to the refinery’s laboratory analysis conducted on real data from February of 2018. As observed in Figure 13, in some hours, up to 14.56 tons of butane does not meet the specification requirements, which forces the refinery to re-inject such subproduct in the distillation process until fulfilling the specification, which ultimately results in a decrease in the amount of butane to sell. However, a prompt prediction of the butane quality in terms of percentage of pentanes allows to readjust the process and reduce the profit losses.

Due to the time-frame needed to reach the new operation point, and considering a conservative approach, only the benefit over 80% of the correctly detected improvable butane is calculated. Thus, in the analysed period and discarding 20% of the detected improvable butane, 258.22 tons of butane that do not fulfil the specification requirements are correctly detected by the proposed soft-sensor. Furthermore, each refinery sets its own sale price for each subproduct. In the refinery from where the data come, the sale price of a ton of butane in February of 2018 was 459.74$. Thus, in the studied two weeks, the profit derived from the online prediction of the subproduct quality with the proposed soft-sensor would be a total of 111,939.35$.

## 5. Conclusions

This work employs real data from a refinery of the Basque Country, and it proposes a soft-sensor to complement the operators’ decision making model by classifying the percentage of pentanes in butane in the bottom of the debutanizer column 400 min in advance based on process information from the top of two naphtha stabilisers columns. The analysis of the different configurations of preprocessing and modelling methods to create a soft-sensor is difficult and time-consuming. Thus, this work proposes an autoML approach that automatically searches for the best configuration among different normalisation, FW preprocessing methods, and commonly employed ML algorithms to select the best configuration among different combinations of methods for a given dataset.

The autoML approach’s preprocessing step employs a novel two-stage methodology that combines normalisation and feature weighting to transform the input space intelligently. The two-stage methodology aims at avoiding features dominance through normalisation methods. FW methods account for the relative importance each feature presents at estimating the real label for improving the classification performance. As proven through this work, the selection of a normalisation method conditions the feature weighting values’ impact at transforming the features, which ultimately conditions the ML algorithm results. Then, three widely utilised normalisation methods, standardisation (ST), min–max normalisation (MM) and median absolute deviation normalisation (MAD), are considered for the two-stage methodology. Two information-theory-based approaches, Random Forest (RF) and Mutual Information (MI), and one statistical method, the Adapted Pearson correlation (P), are applied regarding the feature weighting methods.

As “no free lunch theorem” states, there is no one model that works best for every problem. Thus, for the modelling stage of the autoML approach, seven well-known classification algorithms (QDA, KNN, SVC, RID, LOG, MLP and SGD) are included, among which we select the most appropriate one for the problem at hand.

The autoML approach presented in this work selects the configuration among different preprocessing techniques and ML algorithms that create the most reliable model. For the analysed industrial case, the ST normalisation method with Adapted Pearson correlation-based feature weights and the Logistic regression ML algorithm is selected by the autoML approach as the best configuration to create the soft-sensor. With such configuration, the soft-sensor obtains a precision of 98.925% at predicting the resultant product of improvable quality.

In addition to the classification results obtained at testing the model over two chronologically followed weeks, the estimated profit from applying the developed soft-sensor is presented. Thus, a saving of 111,939.35$ would have resulted from the next two weeks’ classification results.

Along with the promising results obtained by the interpretable proposed approach of combining the two-stage methodology with shallow ML algorithms, in the future, adaptive techniques for online concept drift detection and automatic adaptation of the classification model will be investigated. Furthermore, for the problem at hand, due to the non-stationarity of the time series and the need of selecting a subset of data for the training set, there is no need to remove trend and seasonality. However, as future work, in the case of stationary time series, trend and seasonality removal will be included in the autoML approach. In addition, the proposed autoML has been designed for supervised scenarios. However, in some industrial problems, the labels are difficult to obtain. Thus, in future works, the authors aim to investigate a new approach to handle processes with scarce labels.

## Figures and Tables

**Table 1:** Parameters employed in the grid search for each ML algorithm and the corresponding total number of combinations (Comb) considered in the grid search.

ML	Hyper-Parameters	Comb
QDA	reg_param $\in \{\left\{1,5\right\}\times 10^{-5},\left\{1,5\right\}\times 10^{-4},0.005,0.001,0.05,0.01,0.5,0.1,1\}$	11
KNN	neighbours ∈{5,6,7,…,60}	55
SVC	C ∈{0.0001,0.005,0.001,0.05,0.01,0.5,0.1,5,1,10} γ∈{0.0001,0.001,0.01,0.1,1,10} kernel ∈ {linear, rbf, sigmoid}	180
RID	α∈{0.0001,0.005,0.001,0.05,0.01,0.5,0.1,1,2,4,5,7,10}	13
LOG	C ∈{0.0001,0.005,0.001,0.5,0.01,0.5,0.1,1,5,10}	10
MLP	activation ∈ {identity, logistic, relu} neurons ∈{1,2,3,…,10}	30
SGD	loss ∈{ modified_huber, hinge, squared_hinge, perceptron} α∈{0.00005,0.00001,0.0005,0.0001,0.005,0.001,0.05,0.01,0.5,0.1,1}	44

**Table 2:** *p*-values obtained for the Mann–Kendall test.

	C1_1	C1_2	C1_3	C1_4	C1_5	C1_6	C1_7	C1_8	C1_9	C1_10	C1_11	C1_12	C1_13	C1_14
*p*-value	**0.000**	**0.000**	**0.002**	**0.001**	*0.328*	**0.016**	**0.007**	**0.006**	**0.000**	*0.464*	**0.000**	*0.0513*	**0.004**	*0.373*
	C1_15	C1_16	C1_17	C1_18	C1_19	C1_20	C1_21	C1_22	C1_23	C1_24	C1_25	C1_26	C1_27	C1_28
*p*-value	**0.003**	*0.296*	**0.001**	*0.529*	*0.880*	*0.716*	*0.192*	*0.445*	*0.0661*	**0.001**	*0.220*	*0.686*	*0.201*	*0.196*
	C1_29	C1_30	C1_31	C2_1	C2_2	C2_3	C2_4	C2_5	C2_6	C2_7	C2_8	C2_9	C2_10	C2_11
*p*-value	*0.597*	*0.619*	*0.351*	*0.123*	*0.123*	**0.000**	*0.996*	*0.702*	*0.656*	*0.109*	*0.127*	**0.003**	**0.024**	*0.501*
	C2_12	C2_13	C2_14	C2_15	C2_16	C2_17	C2_18	C2_19	C2_20	C2_21	C2_22			
*p*-value	*0.390*	**0.009**	**0.0001**	*0.364*	*0.448*	*0.443*	*0.728*	*0.725*	*0.983*	*0.445*	*0.952*			

**Table 3:** *p*-values obtained for the Augmented Dickey–Fuller test.

	C1_1	C1_2	C1_3	C1_4	C1_5	C1_6	C1_7	C1_8	C1_9	C1_10	C1_11	C1_12	C1_13	C1_14
*p*-value	**0.000**	**0.008**	**0.028**	*0.241*	*0.185*	*0.100*	*0.087*	**0.006**	*0.340*	*0.083*	*0.176*	*0.275*	*0.397*	**0.000**
	C1_15	C1_16	C1_17	C1_18	C1_19	C1_20	C1_21	C1_22	C1_23	C1_24	C1_25	C1_26	C1_27	C1_28
*p*-value	*0.346*	*0.936*	*0.341*	*0.318*	*0.227*	**0.032**	*0.455*	**0.013**	*0.180*	**0.006**	**0.000**	*0.631*	**0.018**	**0.018**
	C1_29	C1_30	C1_31	C2_1	C2_2	C2_3	C2_4	C2_5	C2_6	C2_7	C2_8	C2_9	C2_10	C2_11
*p*-value	**0.011**	**0.011**	**0.014**	**0.023**	**0.023**	**0.015**	**0.014**	*0.050*	*0.488*	**0.019**	**0.021**	**0.012**	*0.080*	**0.028**
	C2_12	C2_13	C2_14	C2_15	C2_16	C2_17	C2_18	C2_19	C2_20	C2_21	C2_22			
*p*-value	*0.327*	**0.015**	**0.048**	*0.311*	**0.032**	**0.030**	**0.010**	**0.002**	**0.010**	**0.001**	**0.002**			

**Table 4:** *p*-values obtained for the KPSS test.

	C1_1	C1_2	C1_3	C1_4	C1_5	C1_6	C1_7	C1_8	C1_9	C1_10	C1_11	C1_12	C1_13	C1_14
*p*-value	**<** **0.01**	**<0.01**	**<0.010**	**<0.01**	**<0.01**	**<0.01**	**<0.01**	**<0.01**	**<0.01**	**<0.01**	**<0.01**	**<0.01**	**<0.01**	*0.100*
	C1_15	C1_16	C1_17	C1_18	C1_19	C1_20	C1_21	C1_22	C1_23	C1_24	C1_25	C1_26	C1_27	C1_28
*p*-value	**<0.01**	**<0.01**	**<0.01**	**<0.01**	**<0.01**	**<0.01**	**<0.01**	**<0.01**	**<0.01**	**<0.01**	**0.047**	**<0.01**	**<0.01**	**<0.01**
	C1_29	C1_30	C1_31	C2_1	C2_2	C2_3	C2_4	C2_5	C2_6	C2_7	C2_8	C2_9	C2_10	C2_11
*p*-value	**0.049**	**0.033**	**<0.01**	**<0.01**	**<0.01**	**<0.01**	**<0.01**	**<0.01**	**<0.01**	**<0.01**	**<0.01**	**<0.01**	**<0.01**	**<0.01**
	C2_12	C2_13	C2_14	C2_15	C2_16	C2_17	C2_18	C2_19	C2_20	C2_21	C2_22			
*p*-value	**<0.01**	**<0.01**	**0.010**	**<0.01**	**<0.01**	**0.010**	**<0.01**	**<0.01**	**<0.01**	**<0.01**	*0.100*			

**Table 5:** Most relevant features according to each FW method.

FW	Most Relevant Features
P	C2_6, C2_14, C1_13
RF	C1_13, C1_15, C1_16, C1_8
MI	C1_29, C1_15 C1_13, C2_6

**Table 6:** Features with weight value <1/*m* despite the FW method.

C1_2:C1_5, C1_7
C1_9:C1_12, C1_14
C1_15, C1_18, C1_19
C1_21:C1_26, C1_30, C2_11, C2_22

**Table 7:** Difference between the maximum and the mean weight values for each FW method.

	P	RF	MI
$max\left(w_{j}^{*}\right)-\bar{w^{*}}$	0.0175	0.0346	0.0168
$\sum_{j=1}^{m}\left|w_{j}^{*}-1/m\right|$	0.608	0.416	0.395

**Table 8:** Precision reached by each ML algorithm over the raw, normalised and transformed datasets.

Algorithm		Raw	Normalisation			Proposed Methodology								
		X	${\tilde{X}}^{\mathrm{ST}}$	${\tilde{X}}^{\mathrm{MM}}$	${\tilde{X}}^{\mathrm{MAD}}$	${\tilde{X}}_{P}^{\mathrm{ST}}$	${\tilde{X}}_{P}^{\mathrm{MM}}$	${\tilde{X}}_{P}^{\mathrm{MAD}}$	${\tilde{X}}_{\mathrm{RF}}^{\mathrm{ST}}$	${\tilde{X}}_{\mathrm{RF}}^{\mathrm{MM}}$	${\tilde{X}}_{\mathrm{RF}}^{\mathrm{MAD}}$	${\tilde{X}}_{\mathrm{MI}}^{\mathrm{ST}}$	${\tilde{X}}_{\mathrm{MI}}^{\mathrm{MM}}$	${\tilde{X}}_{\mathrm{MI}}^{\mathrm{MAD}}$
QDA		24.414	62.304	64.286	61.340	0.000	38.506	40.909	53.548	72.973	47.689	90.000	0.000	100
KNN		27.551	23.192	40.554	26.359	41.429	39.370	42.529	24.724	38.998	**43.416**	35.057	32.113	37.956
SVC		56.897	0.000	7.368	0.000	22.562	16.068	20.564	52.250	16.333	65.079	21.914	16.071	24.145
RID		81.507	38.517	86.957	51.598	22.938	98.734	54.028	20.511	100	51.598	21.807	96.000	51.835
LOG		90.164	92.029	0.000	100	97.872	0.000	85.714	80.000	100	90.698	92.381	0.000	75.000
MLP	Max	100	82.178	84.647	83.974	88.587	93.878	77.500	78.599	100	75.646	85.976	91.509	75.954
	Mean	34.595	51.622	68.631	58.136	80.558	82.055	73.460	71.364	95.180	71.675	76.591	73.593	72.384
	std	36.386	20.673	11.569	19.943	4.064	5.892	2.066	2.014	3.809	2.049	3.601	6.334	2.106
	Min	0.000	18.171	38.836	18.825	74.717	72.549	69.283	68.910	87.500	65.549	72.852	66.997	68.506
SGD	Max	26.606	46.868	18.929	75.862	71.795	0.000	81.022	41.640	0.000	44.660	23.343	0.000	30.334
	Mean	8.013	42.328	13.236	41.045	34.554	0.000	42.824	37.842	0.000	41.662	18.144	0.000	26.709
	std	6.171	2.028	1.850	13.979	13.019	0.000	10.755	1.365	0.000	1.465	2.359	0.000	1.245
	Min	0.000	38.636	10.304	17.804	15.139	0.000	24.967	35.431	0.000	39.130	12.405	0.000	24.194

**Table 9:** Percentage of precision and recall obtained by each preselected approach for increasing temporal horizons.

	Prediction Horizon					Prediction Horizon			
	1 W	2 W	3 W	4 W		1 W	2 W	3 W	4 W
Precision	0	100	100	100	Precision	96.667	98.734	98.734	98.734
Recall	0	0.277	0.161	0.154	Recall	21.168	21.607	12.52	11.982
(**a**) QDA for $X_{MI}^{MAD}$					(**b**) RID for $X_{P}^{MM}$				
	**Prediction Horizon**					**Prediction Horizon**			
	1 W	2 W	3 W	4 W		1 W	2 W	3 W	4 W
Precision	0	100	100	100	Precision	95.833	96	96	96
Recall	0	3.601	2.087	1.997	Recall	16.788	26.593	15.409	14.747
(**c**) RID for $X_{RF}^{MM}$					(**d**) RID for $X_{MI}^{MM}$				
	**Prediction Horizon**					**Prediction Horizon**			
	1 W	2 W	3 W	4 W		1 W	2 W	3 W	4 W
Precision	97.872	98.925	98.925	97.872	Precision	100	100	100	100
Recall	33.577	25.485	14.767	14.132	Recall	01.460	11.634	06.742	06.452
(**e**) LOG for $X_{P}^{ST}$					(**f**) LOG for $X_{RF}^{MM}$				
